# Literature-Based Drug Repurposing in Traditional Chinese Medicine: Reduced Inflammatory M1 Macrophage Polarization by Jisil Haebaek Gyeji-Tang Alleviates Cardiovascular Disease In Vitro and Ex Vivo

**DOI:** 10.1155/2020/8881683

**Published:** 2020-12-07

**Authors:** Ga-Ram Yu, Seung-Jun Lee, Da-Hoon Kim, Dong-Woo Lim, Hyuck Kim, Won-Hwan Park, Jai-Eun Kim

**Affiliations:** ^1^Department of Diagnostics, College of Korean Medicine, Dongguk University, Dongguk-Ro 32, Goyang 10326, Republic of Korea; ^2^Department of Pathology, College of Korean Medicine, Dongguk University, Dongguk-Ro 32, Goyang 10326, Republic of Korea; ^3^Institute of Korean Medicine, Dongguk University, Dongguk-Ro 32, Goyang 10326, Republic of Korea

## Abstract

Relatively high proportions of proinflammatory M1-like macrophages in tissues may lead to vascular impairment and trigger numerous diseases including atherosclerosis-related cardiovascular disease (CVD). Jisil Haebaek Gyeji-tang (JHGT), a polyherbal decoction, is traditionally used to treat various human ailments including chest pain, angina, and myocardial infarction. In the present study, we investigated the anti-inflammatory effects of JHGT on lipopolysaccharide- (LPS-) stimulated M1 macrophage polarization generated via the mitogen-activated protein kinases (MAPKs) pathway in RAW 264.7 mouse macrophages. The reducing power of JHGT was also investigated using DAF-FA DA in a zebrafish model. JHGT  significantly reduced inflammatory mediator levels, including iNOS, COX2, TNF-*α*, IL-6, and IL-1*β*, as compared with LPS-stimulated controls in vitro and ex vivo. Furthermore, JHGT suppressed the ERK1/2, JNK, and p38 MAPK pathways and reduced p-I*κ*B*α* levels and the nuclear translocation of NF-*κ*B in RAW 264.7 cells. In addition, treatment with JHGT significantly reduced the NO levels in LPS-treated zebrafish larva ex vivo. Our findings show the potent anti-inflammatory properties of JHGT are due to its suppression of MAPK signaling, NF-*κ*B translocation, and M1 macrophage polarization.

## 1. Introduction

Inflammation is complex physiological response to noxious stimuli, such as physical or chemical injuries, or infections including exogenous pathogens such as bacterial lipopolysaccharide (LPS) [1, 2]. However, excessive, uncontrolled proinflammatory responses can cause severe vascular damage and result in atherosclerosis-related cardiovascular diseases (CVD) [3]. Atherosclerosis is the result of a proinflammatory response of arterial walls and can give rise to macrophage polarization via plaque rupture or tissue erosion [4, 5]. The incidence of atherosclerosis-related CVD are steadily increasing in developing and developed countries, and they remain the leading cause of mortality worldwide [6].

Macrophages are the major antigen-presenting cells (APCs) and are well-known to provide crucial molecular alarm signals, which include proinflammatory cytokines, after pathogen invasion [7, 8]. Resident macrophages are activated by stimulation of toll-like receptor (TLR; a primary LPS receptor) to produce proinflammatory cytokines and initiate inflammation in lesions [9, 10]. When inflammatory responses occur in blood vessels, macrophages are polarized into proinflammatory M1 and anti-inflammatory M2 types, and the proinflammatory M1 form predominates in atherosclerotic plaque [11]. Several studies have provided compelling evidence that the presence of a high proportion of M1-type macrophages promotes atherosclerosis [12–14]. Macrophages polarization is also caused by mitochondrial dysfunction, and this too results in excessive productions of inflammatory cytokines and mediators such as interleukins (ILs), tumor necrosis factor-*α* (TNF-*α*), nitric oxide (NO), inducible nitric oxide synthase (iNOS), and cyclooxygenase 2 (COX2) [15, 16]. On contrary to this, M2 phenotype can secrete anti-inflammatory factors such as IL-10 and tumor growth factor *β* (TGF-*β*) and promote tissue remodeling and repairing through collagen formation in CVD events. Therefore, the balance between the M1 and M2 macrophage phenotypes is viewed as a therapeutic target for inflammatory response-related CVD.

Traditional Chinese medicine (TCM) has more than 2,000 years of history, is based on natural products, and is now viewed as attractive safe medicines that provide multicompound-based and multitargeting therapies. Recently, researchers and clinical practitioners in TCM have begun to look into the CVD in molecular concepts, eventually to enhance its diagnosis and treatment [17, 18]. CVD are strongly associated with characteristics of metabolic syndrome, such as obesity, diabetes, and dyslipidemia. In a previous study, we demonstrated that literature-based herbal digestive Sochewan have pharmacological properties and that repurposing of alternative medicine may result in treatments for metabolic syndrome and chronic inflammatory diseases [19]. Also, our another study supports the notion that polyherbal prescription Jwa Kum Whan have potential for the treatment and prevention of metabolic syndrome [20]. Emerging evidences suggest that the promising effect of TCM on CVD is not only on high expectation values but also according to the medicinal effects [21, 22]. However, the key regulatory mechanism responsible for the atheroprotective properties of TCM is not fully understood.

The treatment of chest pain with Jisil Haebaek Gyeji-tang (JHGT) is mentioned in the most famous TCM classic Gum-Gue-Yo-Lak (櫃櫃要略), which states JHGT be administered when “the chest is frustrated and pain is present under the armpits or at the bottom of the heart,” that is, when the symptoms of vascular inflammation-related arteriosclerosis are manifest [23]. Novel approaches are required to treat inflammatory disease, and efforts are being made to discover natural product-derived modulators of macrophage polarization of the treatment of atherosclerosis-related CVD. In a recent study, we reported the protective effects of two similar Gum-Gue-Yo-Lak prescriptions on LPS-stimulated M1 macrophage polarization-induced vascular inflammation [24]. In the present study, we extended our studies by investigating the molecular mechanism responsible for the inhibitory effect of JHGT on the LPS-induced M1 polarization of murine bone marrow-derived macrophages and RAW264.7 cells. In addition, we explored the biological test of NO reduction in LPS-induced zebrafish's larva and confirmed that JHGT treatment could significantly inhibit NO secretion, which might be realized by affecting the regulation of macrophages polarization. Our findings highlighted the anti-inflammatory effects of JHGT, as well as determined the important role of macrophages polarization in the atherosclerosis-related CVD.

## 2. Materials and Methods

### 2.1. Chemicals

Fetal bovine serum (FBS) and penicillin/streptomycin solution were purchased from Invitrogen (Carlsbad, CA, USA) and Dulbecco's Modified Eagle's Medium (DMEM) and RPMI 1640 medium from Hyclone (Logan, UT, USA). Lipopolysaccharide (LPS) and other reagents were obtained from Sigma Chemicals (St. Louis, MO, USA). Mouse recombinant IFN gamma was supplied by GenScript (Piscataway, NJ, USA). ELISA kits for IL-1*β*, IL-6, and TNF-*α* were obtained from R&D Systems (Minneapolis, MN, USA). Primary antibodies for lamin B, iNOS, COX2, and *β*-actin and horseradish peroxidase- (HRP-) conjugated secondary antibodies were purchased from Santa Cruz (Dallas, TX, USA). Primary antibodies, including phosphor-extracellular signal-regulated kinases (*p*-ERK), phosphor-c-Jun N-terminal kinases (p-JNK), phosphor-p38 mitogen-activated protein kinases (p-p38), p-I*κ*B-*α*, and p-NF-*κ*B (p-p65) were obtained from Cell Signaling Technology (Beverly, MA, USA). The oligonucleotide primers used in real-time qPCR were supplied by Macrogen (Seoul). DAF-FM DA (4-amino-5-methylamino-2′, 7′-difluorofluorescein diacetate) was supplied by Sigma Chemicals (St. Louis, MO, USA).

### 2.2. Herbal Formula Preparation

The prescription used in this study was prepared by subjecting a JHGT herbal mixture (Fructus Ponciri Seu Aurantii Immaturus: Bulbus Allii Macrostemi: Ramulus Cinnamomi: Cortex Magnoliae Officinalis: Fructus Trichosanthis = 45 g: 300 g: 40 g: 160 g: 25 g) to aqueous reflux extraction at 95°C for 3 h as described in an original text. The herbal mixture was purchased from Human herb (Gyeongsangbuk-do, South Korea). The hot water extract obtained was filtered and concentrated using a rotary evaporator (Buchi, Flawil, Switzerland) at 95°C and freeze-dried to obtain JHGT (3.51%, w/w), as previously described with slight modification [25]. Voucher specimens were deposited at the Institute of Korean Medicine, Dongguk University (DG-DIA017 W).

### 2.3. Animals and Culture Cells

Bone marrow-derived macrophages were isolated from 8-week-old C57BL/6J male mice obtained from Orient Bio (Gyeonggi-do, South Korea). Cells were isolated as previously described [26]. Briefly, mice were anesthetized with a Zoletil and Rompun mixture, and upper and lower parts of femurs were excised. RPMI 1640 medium was then passed through femur segments (from top to bottom) using a syringe, and the cells were obtained by centrifuging the cell suspension at 3,000 rpm for 8 min. Macrophages were then plated in RPMI-1640 containing 10% FBS in 6-well cell culture plates, incubated until 90 confluent, differentiated with the recombinant mouse macrophage colony stimulating factor (M-CSF) (20 ng/mL) for 72 h, and treated with LPS for another 48 h to induce M1 polarization. The protocols used in animal experiments were approved beforehand by the Ethics Committee of Dongguk University (IACUC-2020-030–1).

Raw264.7 cells (a murine macrophage cell line) were purchased from the Korea Cell Line Bank (KCLB, Seoul). Cells were cultured in DMEM supplemented with 10% FBS containing 100 U/mL penicillin and 100 *μ*g/mL streptomycin at 37°C in a humidified 5% CO_2_ environment.

### 2.4. Cell Viability Assay

Cell viabilities were determined using the EZ-Cytox cell viability assay kit (Daeil Lab Service, Seoul) according to the manufacturer's instructions with slight modification [27]. Briefly, mouse bone marrow-derived macrophages or RAW264.7 cells were maintained at 70–80% confluence and then seeded at a density of 1.2 × 10^5^ cells/mL in 96-well plates. After 24 h of incubation, the medium was changed to FBS-free DMEM containing serially diluted JHGT (0–200 *μ*g/mL). After incubation for another 24 h, 10 *μ*l of EZ-Cytox reagent was added to each well, and cells were incubated at 37°C in the humidified 5% CO_2_ incubator for 1 h. Optical densities (ODs) were measured at 450 nm using a microplate reader (VersaMax, Molecular Devices, CA, USA).

### 2.5. Nitrite Assay

Griess reagent was used to investigate the inhibitory effect of JHGT  on LPS-induced nitrite levels, as we previously described [28]. Briefly, RAW264.7 cells were seeded in triplicate on 6-well culture plates at 1.2 × 10^5^ cells/mL and incubated at 37°C in the humidified 5% CO_2_ incubator for 24 h. Cells were then pretreated with various concentrations of JHGT in the absence or presence of LPS (1 *μ*g/mL) for 24 h. Supernatants were collected and mixed with Griess reagent. ODs were measured at 570 nm, and nitrite concentrations were calculated using a standard curve.

### 2.6. Preparation of Nuclear and Cytosolic Fraction

Nuclear and cytosolic proteins were separated using the Nuclear and Cytoplasmic Extraction Reagents kit from Thermo Fisher Scientific. Briefly, RAW 264.7 macrophages were seeded on 60 mm cell cultures dishes at 1.2 × 10^5^ cells/mL and pretreated with JHGT  at 25–100 *μ*g/mL for 1 h. Cells were then stimulated with LPS (1 *μ*g/mL) for 24 h. The nuclear translocation of NF-*κ*B from cytoplasm was assessed by Western blot.

### 2.7. Immunoblot Assay

Protein levels of iNOS, COX2, MAPKs (ERK1/2, JNK, and p38), NF-*κ*B (I*κ*B*α* and p65), lamin B, and *β*-actin were determined by Western blotting as previously described with slight modification [29]. Briefly, cells were washed with ice-cold DPBS and lysed with radio immunoprecipitation assay buffer (Thermo Fisher Scientific, Rockford, IL, USA) containing protease inhibitor and phosphatase inhibitor cocktail (GenDEPOT, Barker, TX, USA). Protein concentrations were measured using the BCA kit (Thermo Fisher Scientific). Protein lysates (30 *μ*g) were loaded into 10% SDS-PAGE gels, electrophoresed, and transferred to PVDF membranes at 100 V for 70 min using an electrophoretic transfer cell (Bio-Rad, Hercules, CA, USA). Membranes were blocked with 5% BSA in TBS/T (containing 0.1% Tween 20) for 1 h, and blots were incubated with primary antibodies (diluted at 1 : 1500 in TBS/T containing 3% BSA) at 4°C overnight with gentle shaking. After washing with TBS/T, membranes were incubated with secondary antibodies (diluted at 1 : 3000 in TBS/T) at room temperature for 2 h. Blots were detected using a Western blot imaging system (Fusion Solo, Vilber Lourmat, Collegien, France), and proteins were visualized using a chemiluminescent ECL buffer (Super Signal West Pico, Thermo Fisher Scientific).

### 2.8. Quantitative Real-Time Polymerase Chain Reaction

The expression levels of proinflammatory mediators were determined by quantitative real-time polymerase chain reaction (qPCR). Total RNA was isolated from RAW264.7 cells and bone marrow macrophages using TRIzol reagent (Thermo Fisher Scientific) according to the manufacturer's instructions [30]. Briefly, reverse transcription was performed using AccuPower RT PreMix (Bioneer, Daejeon, South Korea) and oligo deoxythymine (dT) 18 primers (Invitrogen, Carlsbad, CA, USA). Amplification of primer-specific binding cDNA was performed using a LightCycler 480 PCR system (Roche, Basel, Switzerland). Reactants include 10 *μ*L of SYBR Green Master mixture (Roche, Switzerland), 8 *μ*L of ultrapure water, 10 pmol/*μ*L of primer, and 1 *μ*L of templated cDNA. Amplification was performed using the following schedule: denaturation at 95°C for 10 min, followed by 45 amplification cycles (denaturation at 95°C for 10 s and annealing at 50∼60°C for 5 min). Threshold cycle values (Ct value) were used to quantify PCR products. The primers used were as follows: iNOS forward, 5′-GAGACAGGGAAGTCTGAAGCAC-3′, reverse, 5′- CCAGCAGTAGTTGCTCCTCTTC-3′; COX2 forward, 5′- GCGACATACTCAAGCAGGAGCA-3′, reverse, 5′- AGTGGTAACCGCTCAGGTGTTG-3′; TNF-*α* forward, 5′-AAGCCTGTAGCCCACGTCGTA-3′, reverse, 5′- GGCACCACTAGTTGGTTGTCTTTG-3′; IL-1*β* forward, 5′- CTGAACTCAACTGTGAAATGCCA-3′, reverse, 5′- AAAGGTTTGGAAGCAGCCCT-3′; IL-6 forward, 5′- CCACTTCACAAGTCGGAGGCTTA-3′, reverse, 5′- GCAAGTGCATCATCGTTGTTCATAC-3′, and *β*-actin forward 5′- GCAAGTGCTTCTAGGCGGAC-3′, and reverse 5′- AAGAAAGGGTGTAAAACGCAGC-3′ (*β*-actin was used as the internal control). Results were normalized by dividing the Ct values of genes by that of *β*-actin. Data were acquired using Roche LightCycler 480 software (Roche Applied Science, USA).

### 2.9. Immunofluorescence Assay

To follow the nuclear localization of NF-*κ*B, RAW264.7 cells were grown on Lab-Tek II chamber slides (Nalge Nunc, IL, USA) as previously described with slight modification [31]. Briefly, cells were fixed in 4% formaldehyde for 10 min, permeabilized with 0.1% Triton X-100 for 10 min at room temperature, blocked with 1% BSA for 1 h, and labeled with 2 *μ*g/mL of primary antibody for 3 h. NF-kB in cytoplasm and nuclei was detected by treating cells with PBS containing 2 *μ*g/mL of FITC and 0.2% BSA for 45 min. Nuclei were stained with DAPI (Vector Laboratories, CA, USA) and observed and photographed under a fluorescence microscope (BX50, Olympus, Japan).

### 2.10. ELISA Analysis

Concentrations of inflammatory cytokines, that is, IL-1*β*, IL-6, and TNF-*α* in cell culture supernatants, were quantified using Quantikine ELISA kits (R&D Systems, Inc. Minneapolis, MN, USA) as according to the manufacturer's instructions [32]. Briefly, RAW264.7 cells were seeded at 1.2 × 10^5^ cells/mL in 24-well plates and pretreated with JHGT  at 25, 50, and 100 *μ*g/mL for 1 h. Cells were then stimulated with LPS (1 *μ*g/mL) for 24 h. Culture media were then collected, and the concentrations of IL-1*β*, IL-6, and TNF-*α* were measured using an ELISA kit.

### 2.11. Nitrite Determination in Zebrafish

Synchronized zebrafish embryos were collected, pipetted at 20 embryos/well into six-well plates containing 2 mL of embryo medium E2 buffer (5 mM NaCl, 0.17 mM KCl, 0.33 mM MgSO_4_, 0.33 mM CaCl_2_, and 5% methylene blue) at 7–9 h postfertilization, and incubated with or without of JHGT (100 *μ*g/mL) for 1 h. Embryos were then stimulated with LPS (10 *μ*g/mL) for 24 h at 28.5°C and transferred to fresh embryo medium E2 buffer. NO levels in LPS-stimulated zebrafish were measured using the fluorescent probe dye, 4-amino-5-methylamino-2′, 7′-difluorofluorescein diacetate (DAF-FM DA), which reacts with NO to produce highly fluorescent triazole derivatives. After 7 h in embryo medium E2 buffer, LPS-stimulated zebrafish larvae were transferred into 96-well plates and treated with DAF-FM DA solution (2.5 *μ*M) for 1 h in the dark at 28.5°C, rinsed in fresh embryo medium, and fixed in 4% formaldehyde. The fluorescence intensities of individual larvae were assessed using a fluorescence microscope (BX50, Olympus, Japan) [33]. LPS-stimulated zebrafish larvae were transferred into 96-well plates and treated with DAF-FM DA solution (2.5 *μ*M) for 1 h in the dark at 28.5°C. Following incubation, zebrafish larvae were rinsed in fresh zebrafish embryo medium and fixed in 4% formaldehyde solution prior to observation. The fluorescence intensity of individual zebrafish larvae was quantified using a fluorescence microscope (BX50, Olympus, Japan).

### 2.12. Statistical Analyses

Experimental data were analyzed using GraphPad Prism version 5.0 software (GraphPad, La Jolla, CA, USA). Standard curves were constructed using Excel and PowerPoint (Microsoft, Redmond, WA, USA). Analysis of variance and one-way ANOVA with Dunnett's multiple comparison tests were used to determine the significances of differences between samples. Results are presented as means ± SDs, and *P* values of <0.05 were considered statistically significant.

## 3. Results

### 3.1. Effects of JHGT on RAW264.7 Viability and NO Production

**Figure 1:**
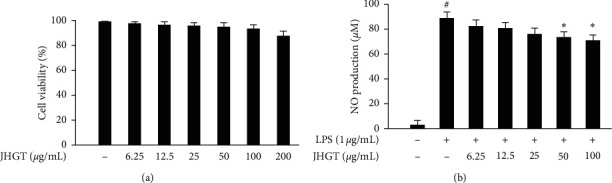
Effect of JHGT on RAW264.7 cell viability and NO production. (a) RAW264.7 cells were incubated for 24 h with JHGT at 0–200 *μ*g/mL. Cell viabilities were measured using an EZ-Cytox assay kit. Results are expressed in percentages of nontreated controls. (b) RAW264.7 cells were pretreated with JHGT for 1 h and then cotreated with JHGT and LPS (1 *μ*g/mL) for 24 h. NO concentrations were measured using Griess reagent. Results are presented as the means ± SDs of three different experiments. ^##^*P* < 0.01 versus nontreated controls, and ^*∗*^*P* < 0.05 and ^*∗∗*^*P* < 0.01 versus LPS-treated controls.

To evaluate the effect of JHGT, RAW 264.7 cell viabilities were measured using the EZ-Cytox assay kit. Cells were incubated with various concentrations of JHGT (6.25, 12.5, 25, 50, 100, or 200 *μ*g/mL) for 24 h, and the cell viabilities observed were 98.51, 97.28, 96.5, 95.81, 94.33, and 88.64%, respectively (Figure 1(a)). When mouse macrophages were pretreated with JHGT and then stimulated with LPS, JHGT dose-dependently reduced LPS-induced NO production (Figure 1(b)). Subsequent experiments were performed using JHGT at concentrations of 25, 50, or 100 *μ*g/mL.

### 3.2. JHGT Inhibited iNOS and COX2 Expressions in LPS-Stimulated RAW264.7 Cells

**Figure 2:**
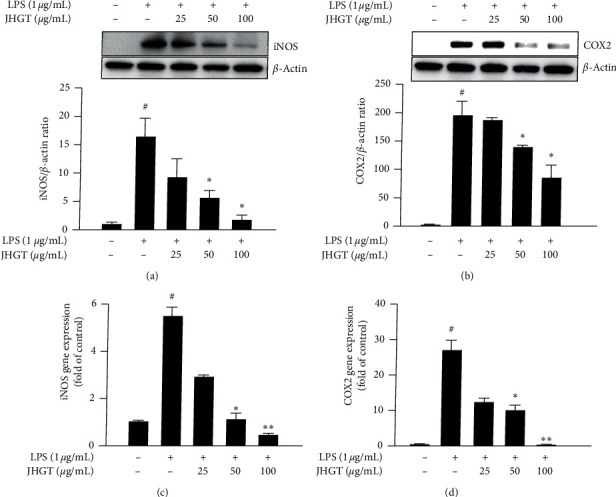
JHGT inhibited LPS-induced upregulations of iNOS and COX2 in RAW264.7 cells. RAW264.7 cells were pretreated with JHGT for 1 h and then treated with LPS (1 *μ*g/mL) for 24 h. (a-b) Relative expressions of inflammatory iNOS and COX2 by Western blot. (c-d) Relative gene expression levels of iNOS and COX2 by real-time PCR. qPCR data were normalized by dividing the Ct values of genes by that of *β*-actin. Results are presented as the means ± SDs of three different experiments. ^##^*P* < 0.01 versus nontreated normal controls, and ^*∗*^*P* < 0.05 and ^*∗∗*^*P* < 0.01 versus LPS-treated controls.

Because iNOS is stimulated under inflammatory conditions and produces NO, we assessed the expressions of iNOS and COX2 in LPS-stimulated macrophages pretreated with JHGT. JHGT pretreatment significantly and dose-dependently inhibited the LPS-induced production of iNOS and obviously suppressed COX2 protein levels (Figures 2(a) and 2(b)). Furthermore, JHGT pretreatment at 50 or 100 *μ*g/mL significantly reduced the LPS (1 *μ*g/mL) induced upregulations of the iNOS and COX2 genes (Figures 2(c) and 2(d)).

### 3.3. JHGT Reduced LPS-Induced Proinflammatory Cytokine Increases in RAW264.7 Cells

**Figure 3:**
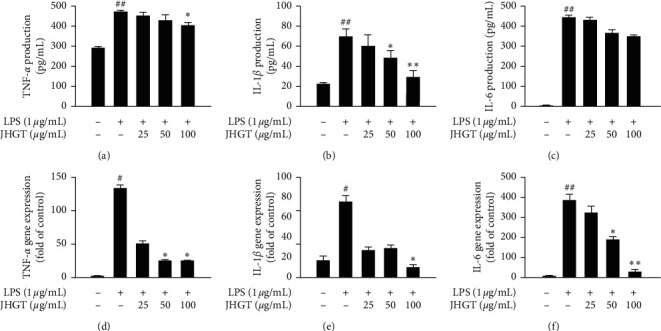
JHGT  reduced LPS-induced increases in proinflammatory cytokine levels in RAW264.7 cells. Cells were pretreated with JHGT  for 1 h and then treated with LPS (1 *μ*g/mL) for 24 h. (a–c) Relative expressions of TNF-*α*, IL-1*β*, and IL-6 as determined by ELISA. (d–f) Relative expressions of TNF-*α*, IL-1*β*, and IL-6 as determined by qPCR. Results are presented as the means ± SDs of three different experiments. ^##^*P* < 0.01 versus nontreated normal controls, and ^*∗*^*P* < 0.05 and ^*∗∗*^*P* < 0.01 versus LPS-treated controls.

ELISA and qPCR were used to investigate the effects of JHGT  on proinflammatory cytokine levels. Pretreatment with JHGT at 50 or 100 *μ*g/mL significantly inhibited LPS-induced increases in TNF-*α*, IL-1*β*, and IL-6 in RAW 264.7 cells (Figures 3(a)–3(c)). qPCR results confirmed that JHGT markedly suppressed the gene expressions of TNF-*α*, IL-1*β*, and IL-6 in LPS-stimulated RAW 264.7 cells (Figures 3(d)–3(f)).

### 3.4. JHGT Regulated MAPK Activity in LPS-Stimulated RAW 264.7 Cells

**Figure 4:**
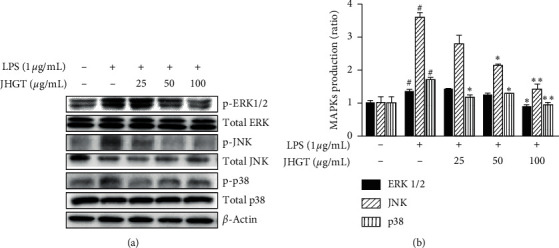
JHGT   regulated MAPK activity in LPS-stimulated RAW 264.7 cells. Cells were pretreated with JHGT  for 1 h and then treated with LPS (1 *μ*g/mL) for 24 h. (a) Relative MAPK expressions as determined by Western blot. (b) Band intensities were measured by densitometry and normalized versus the intensities of total forms and *β*-actin. Results are presented as the means ± SDs of three different experiments. ^##^*P* < 0.01 versus normal nontreated controls, and ^*∗*^*P* < 0.05 and ^*∗∗*^*P* < 0.01 versus LPS-treated controls.

Several studies have suggested that MAPK signal cascades, such as those of ERK1/2, JNK, and p38 MAPKs, play important roles in the inflammatory responses induced by LPS in macrophages [34, 35]. Western blot analysis was used to confirm the inhibitory effect of JHGT  on the activations of MAPK signals by LPS. At 1 *μ*g/mL, LPS markedly increased the phosphorylations of ERK1/2, JNK, and p38 MAPK, and pretreatment with JHGT inhibited these effects of LPS (Figure 4(a)) and dose-dependently reduced the phosphorylation of JNK (Figure 4(b)).

### 3.5. JHGT Suppressed the Nuclear Translocation of NF-*κ*B in RAW 264.7 Cells

**Figure 5:**
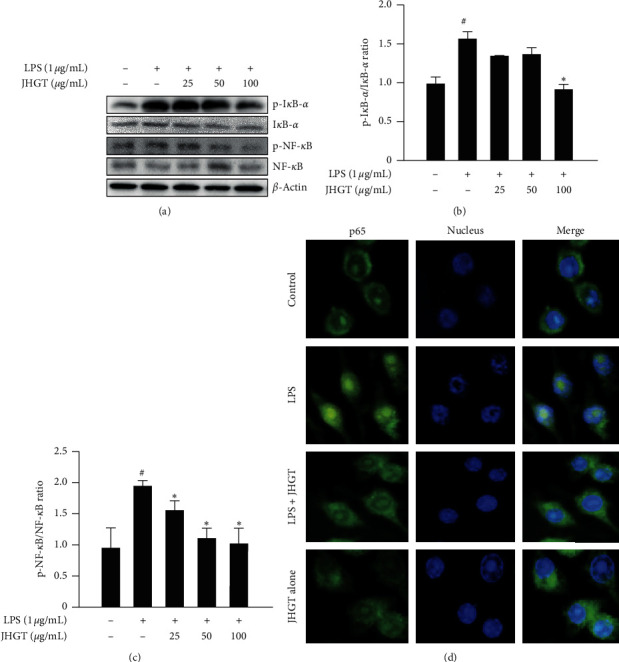
JHGT suppressed NF-*κ*B signal translocation in RAW 264.7 cells. Cells were pretreated with JHGT for 1 h and then treated with LPS (1 *μ*g/mL) for 24 h. (a) Relative phosphorylations of intracellular NF-*κ*B and I*κ*B*α* and their total protein levels as determined by Western blot. (b–d) The nuclear translocation of NF-*κ*B (p65) was visualized by immunofluorescence microscopy. Results are presented as the means ± SDs of three different experiments. ^##^*P* < 0.01 versus normal nontreated controls, and ^*∗*^*P* < 0.05 and ^*∗∗*^*P* < 0.01 versus LPS-treated controls.

Western blot showed that JHGT significantly inhibited the LPS-induced phosphorylations of NF-*κ*B (Figures 5(a)–5(c)) and I*κ*B-*α* (Figure 5(b)), and when JHGT pretreated RAW 264.7 cells were stimulated with LPS, immunofluorescence microscopy showed that JHGT reduced the LPS-induced nuclear translocation of p65 (Figure 5(d)). However, JHGT (100 *μ*g/mL) alone had no obvious effect on translocation of p65.

### 3.6. JHGT Inhibited Inflammatory Mediator Responses Induced by LPS Plus INF*γ* Cotreatment and Regulated M1 Polarization in Mouse Bone Marrow-Derived Macrophages

**Figure 6:**
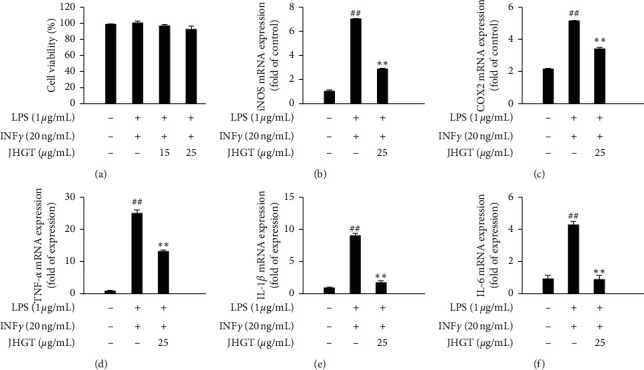
JHGT inhibited inflammatory mediator inductions by LPS plus INF*γ* cotreatment and regulated M1 polarization responses in mouse bone marrow macrophages. (a) Cytotoxic effect of JHGT on M1 polarized from bone marrow-derived macrophages. Cells were incubated with various concentrations of JHGT for 24 h. Results are expressed as percentages of nontreated controls. (b–f) Relative gene expression levels in bone marrow-derived M1-like macrophages as determined by real-time PCR. Results are presented as the means ± SDs of three different experiments. ^##^*P* < 0.01 versus normal nontreated controls, and ^*∗*^*P* < 0.05 and ^*∗∗*^*P* < 0.01 versus LPS plus INF*γ* cotreated controls.

To investigate the influence of JHGT on inflammatory mediators and M1 polarization, we used mouse bone marrow-derived macrophages. Cells were treated with 15 or 25 *μ*g/mL of JHGT in the absence or presence of LPS (1 *μ*g/mL) plus INF*γ* (20 ng/mL) for 24 h (Figure 6(a)). Cotreatment with IFN*γ* and LPS potently induced M1 inflammatory mediators, but pretreatment with JHGT at 25 *μ*g/mL significantly reduced the mRNA levels of iNOS, COX2, TNF-*α*, IL-1*β*, and IL-6 (Figures 6(b)–6(f)).

### 3.7. JHGT Decreases NO Production in Zebrafish

**Figure 7:**
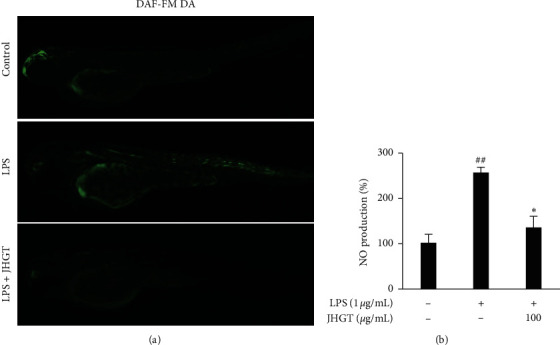
Effect of JHGT on LPS-induced NO production in zebrafish embryos. Zebrafish embryos were pretreated with JHGT (100 *μ*g/mL) for 1 h and then exposed to LPS (10 *μ*g/mL) for 24 h. (a) NO levels were measured using DAF-FM-DA. (b) Results were quantified using Image J software and are presented as the means ± SDs of three different experiments. ^##^*P* < 0.01 versus normal nontreated controls, and ^*∗*^*P* < 0.05 versus LPS-treated controls.

Zebrafish are often used as an in vivo model because like mammals, they have innate and acquired immune systems. We investigated whether JHGT inhibits LPS-induced NO production in zebrafish using the fluorescent probe DAF-FM (Figure 7(a)). LPS treatment stimulated NO production by 241% in zebrafish, and JHGT pretreatment significantly reduced this LPS-induced increase in NO production (Figure 7(b)).

## 4. Discussion

“Geum-Gwe-Yo-Ryak (櫃匱要略),” the oldest pharmacological Bible, recorded to Kwaru Haeback Paekju (KHP), Kwaru Haeback Banha (KHB), and Jisil Haebaek Gyeji-tang (JHGT), treated for chest pains in Chapter 9 [36]. In particular, KHP, KHB, and JHGT were constitutional to two same herbs, that is, *Trichosanthes kirilowii* Maxim. (Trichosanthis Fructus) and *Allium macrostemon* Bunge. (Allii Macrostemonis Bulbus). *A. macrostemon*, a major constituent of JHGT, has been reported to have a therapeutic effect on acute myocardial ischemia via reducing inflammatory responses [37]. Furthermore, *A. macrostemon* and *T. kirilowii* are among the top 10 natural products for the treatment of coronary artery diseases [38]. Recently, a study undertaken by our group showed that both KHP and KHB alleviate LPS-induced inflammatory responses in RAW264.7 cells. In a previous study, we also found that these prescriptions have beneficial effects on atherosclerotic-related CVD lesions, which are known to be regulated by macrophage polarization [24].

*Cinnamomi cassia* Presl. (Cinnamomi Ramulus), which was another JHGT constitutional herb, has been treated with the literature-based weak chest pain or prodromal symptoms. *C. cassia* is well known and commonly used in TCM, and it has also been reported to have a wide range of pharmacological properties, that is, to reduce oxidative stress and inflammation and protect the cardiovascular system [36, 39, 40]. Furthermore, *C. cassia* contains many known anti-inflammatory compounds such as cinnamaldehyde, cinnamyl acetate, and cinnamyl alcohol, which reduce the mRNA expressions of inflammatory cytokines (e.g., IL-1*β*, IL-6, and TNF-*α*) in LPS-induced RAW 264.7 macrophages [41, 42].

Nitric oxide (NO) is an important free radical and is induced by endotoxins such as LPS. Under inflammatory conditions, iNOS generates excessive nitric oxide (NO) in blood vessels, which in turn, increases tissue levels of inflammatory mediators such as COX2, TNF-*α*, IL-1*β*, and IL-6 [43]. IL-6 is well known to enhance inflammatory signal transduction, which leads to the upregulations of proinflammatory signals such as ERK1/2, JNK, and p38 kinase [44]. Therefore, reducing NO production is regarded as one of the best ways of preventing the progression of treating inflammatory diseases such as atherosclerosis-related CVD. Our results show that JHGT significantly decreases NO levels in LPS-induced RAW264.7 cells, and our ex vivo results show that JHGT reduces NO levels in INF-*γ* plus LPS cotreated mouse bone marrow-derived macrophages. In addition, in vitro results show that JHGT dose-dependently and significantly downregulated levels of inflammatory mediators, including COX2, TNF-*α*, IL-1*β*, and IL-6.

In the context of inflammatory response in macrophages, LPS can also activate inflammatory cell signal pathways via MAPKs and NF-*κ*B [45]. MAPKs are well-known serine/threonine protein kinases and are responsible for signal transduction under various proinflammatory conditions [46]. Furthermore, nuclear factor kappa B (NF-*κ*B) is modulated by inflammatory mediators, and this is followed by activation of the MAPK signaling pathway [47, 48]. Modulation of DNA binding activity of NF-*κ*B is regulated by the phosphorylation and degradation of I*κ*B [49]. Therefore, blocking these MAPK and NF-*κ*B cell signaling cascades offers a potential means for treating inflammation-related atherosclerotic development. We also investigated the effects of JHGT on the MAPK signaling pathway and nuclear translation of NF-*κ*B in LPS-stimulated macrophages. When RAW264.7 cells were treated with LPS (1 *μ*g/mL), the phosphorylations of ERK, JNK, and p38 MAPK and the nuclear translocation of p65 increased, but pretreatment with JHGT significantly reduced these effects.

Polarized macrophages may be of M1 (proinflammatory) or M2 (anti-inflammatory) type, and M1 macrophages play a critical role in adaptive immune response in blood vessels [50]. However, excessive, uncontrolled proinflammatory response causes mitochondrial dysfunction and endothelial damage and results in an imbalance of macrophages polarization toward the M1 phenotype [51]. Previous study has suggested that unmoderated macrophage M1 polarization complicates inflammatory responses and leads to atherosclerosis-related CVD [52]. Several pioneering studies suggested that immoderated macrophage M1 polarization complicates inflammatory responses, which leads to CVD-related atherosclerosis [53, 54]. In the present study, we found that CD86 acted as a marker of M1 macrophage polarization (data not shown). CD86 is expressed on activated APCs (antigen-presenting cells) in response to pathogens and leads to the stimulation of proinflammatory cell signaling pathways such as those controlled by MAPK and NF-*κ*B. Furthermore, interferon gamma (INF*γ*) and LPS act synergistically on macrophages and ultimately trigger M1 macrophage polarization. In the present study, using CD86 as a marker, JHGT pretreatment was found to significantly reduce the proportion RAW264.7 cells and bone marrow-derived murine macrophages exhibiting the M1 phenotype. These observations indicate that JHGT reinforces the anti-inflammatory pathway and causes the repolarization of macrophages of the M1 phenotype under pathogen-induced inflammatory conditions.

Animal research studies support that the zebrafish (*Danio rerio*) model provides a valuable means for studying human disease and discovering new drugs. Recently, the zebrafish was found to share 70% functional similarity to humans at the gene level [55, 56]. We observed by fluorescence microscopy that JHGT strongly reduced NO levels in our LPS-induced zebrafish larva model ex vivo. The probable explanation of this result is ex vivo study macrophages driven by repolarization; thus, we suggest further experiments be undertaken to identify the molecular mechanism of atherosclerosis-related CVD and the way JHGT influences this mechanism.

## 5. Conclusions

In summary, our findings provide insight into the possible repositioning of a traditional medication for the treatment of atherosclerosis-related CVD. Importantly, JHGT was found to reduce M1 macrophage polarization, which appeared to be responsible for its anti-inflammatory activities. These results suggest that JHGT be considered a potential treatment for atherosclerosis-related CVD.

## Data Availability

The data used to support the findings of this study are included within the article.

## Abbreviations

NO: Nitric oxide
LPS: Lipopolysaccharide
IL-1*β*: Interleukin-1 beta
TNF-*α*: Tumor necrosis factor alpha
iNOS: Inducible nitric oxide synthase
COX2: Cyclooxygenase 2
MAPKs: Mitogen-activated protein kinases
NF-*κ*B: Nuclear factor kappa B
CD86: Cluster differentiation 86
IFN*γ*: Interferon gamma.
